# Can baseline quality of life scores predict for morbidity and survival after CRS and HIPEC: a prospective study of 151 patients

**DOI:** 10.1515/pp-2021-0148

**Published:** 2022-04-04

**Authors:** Claramae Shulyn Chia, Chin-Ann Johnny Ong, Hong-Yuan Zhu, Cindy Lim, Jolene Si Min Wong, Grace Hwei Ching Tan, Melissa Ching Ching Teo

**Affiliations:** Department of Sarcoma, Peritoneal and Rare Tumours (SPRinT), Division of Surgery and Surgical Oncology, National Cancer Centre Singapore, Singapore, Singapore; Department of Sarcoma, Peritoneal and Rare Tumours (SPRinT), Division of Surgery and Surgical Oncology, Singapore General Hospital, Singapore, Singapore; SingHealth Duke-NUS Oncology Academic Clinical Program, Duke-NUS Medical School, Singapore, Singapore; SingHealth Duke-NUS Surgery Academic Clinical Program, Duke-NUS Medical School, Singapore, Singapore; Laboratory of Applied Human Genetics, Division of Medical Sciences, National Cancer Centre Singapore, Singapore, Singapore; Institute of Molecular and Cell Biology, A*STAR Research Entities, Singapore, Singapore; Division of Clinical Trials and Epidemiological Sciences, National Cancer Centre Singapore, Singapore, Singapore

**Keywords:** baseline QoL, cytoreductive surgery (CRS), hyperthermic intraperitoneal chemotherapy (HIPEC), peritoneal carcinomatosis

## Abstract

**Objectives:**

Various studies have shown that good quality of life (QoL) can be achieved after cytoreductive surgery (CRS) and hyperthermic intraperitoneal chemotherapy (HIPEC). There is prognostic value of baseline QoL in post-operative outcome in Western setting. Our prospective study aims to validate these observations and elucidate clinical factors that predict poorer QoL in Asian peritoneal carcinomatosis patients.

**Methods:**

European Organization for Research and Treatment of Cancer Core Quality of Life Questionnaire was administered to patients before CRS and HIPEC and thereafter at 3, 6 and 12 months.

**Results:**

A total of 151 patients underwent 155 surgeries. Four hundred and seventy two questionnaires were completed. Median disease-free survival (DFS) was 16.5 months. Three year DFS and overall survival (OS) were 24.0% and 73.0% respectively. Post-operative global health status significantly increased at 3, 6 and 12 months. The decreases in functional scales recovered to baseline by 1-year post-surgery. Peritoneal carcinomatosis index (PCI), presence of stoma, peritonectomy duration, death within one year, post-operative complication and length of SICU stay negatively influenced QoL. Complication rates were higher in patients with lower global health status, physical and role functioning scores and higher symptom summary scores at baseline. Lower social functioning score, and higher pain, dyspnoea and symptom summary scores at baseline were significantly associated with poorer OS.

**Conclusions:**

Various clinical factors can help us predict a patient’s QoL after surgery. Several baseline factors were also able to predict morbidity and survival. Going forward, we can use these factors to help us better select patients who will have a greater benefit from CRS and HIPEC.

## Introduction

Patients with peritoneal surface malignancies were once considered terminal and treated palliatively with chemotherapy or best supportive care. With the advent of cytoreductive surgery (CRS) and hyperthermic intraperitoneal chemotherapy (HIPEC) in the early 1990s, some of these patients now have a possibility of cure. CRS and HIPEC is now the standard of care for patients with pseudomyxoma peritonei, peritoneal mesothelioma and limited peritoneal carcinomatosis (PC) from colorectal cancer. It has enabled prolonged survival in those with PC from ovarian cancer and continues to be investigated for its role in gastric PC. Multiple studies have shown that the morbidity and mortality of performing CRS and HIPEC are comparable to other major abdominal surgeries, as the improvement in surgical technique and anaesthetic ability continues [1]. In experienced centres, the mortality ranges from 0 to 8% while the morbidity ranges from 25 to 41%.

In spite of the improved survival and acceptable morbidity and mortality of CRS and HIPEC, concerns about the quality of life (QoL) for survivors remain. Prospective studies have shown that QoL can return to baseline for long-term survivors [2], [3], [4], [5], [6]. Although there is an initial decrease in scores just after surgery, scores increase to baseline levels within 12 months. However, most prospective studies investigating QoL in PC patients who underwent CRS and HIPEC were performed in Western setting [7], [8], [9] and their results may not reflect Asian population’s QoL as culture and ethics difference can affect the perception of QoL [10]. Thus, in this prospective study, we sought to validate these observations in Asian population.

Patient selection based on the predicted risk of the post-operative morbidity and mortality is imperative for planning treatment. Numerous studies have demonstrated the prognostic value of patients’ baseline QoL for overall survival (OS) and post-operative complication across various cancer types [11], [12], [13]. However, limited studies examine the prognostic value of QoL in survivors of patients who underwent CRS and HIPEC for PC from various primary sites. Two studies conducted by the same group in USA [8, 9] reported baseline QoL measured by FACT-C and SF-36 predicted morbidity and mortality following CRS and HIPEC. EORTC-QLQ-C30 is also a widely used questionnaire for measuring cancer patients’ QoL. It assesses cognitive function and financial impact of disease which are not covered by FACT-C. Additionally, it is different from FACT-C in items phrasing: FACT-C is presented as statements while EORTC-QLQ-C30 is presented as questions [14]. Thus, in this prospective study, we sought to assess the association between baseline QoL measured by EORTC-QLQ-C30 and post-operative outcomes in survivors of PC patients who underwent CRS and HIPEC. We also elucidated clinical factors that predict poorer QoL and assessed the impact of CRS and HIPEC on QoL.

## Materials and methods

From February 2012 to April 2017, consecutive patients who underwent CRS and HIPEC at National Cancer Centre Singapore for PC from various pathologies were included. Patients were eligible for surgery if they did not have any other metastatic disease and were of Eastern Cooperative Group (ECOG) performance status 0 or 1. The study complied with Helsinki Declaration and was approved by SingHealth Centralised Institutional Review Board. Eligible patients were consented before surgery.

### Data collection

EORTC-QLQ-C30 was used. We included QLQ-CR29 and QLQ-OV28 for patients with colorectal and ovarian primary cancers, respectively. Both English and Chinese versions of questionnaire were used. To ensure standardised interpretation of questionnaire, our research assistants, who were not involved in patients’ care, administered questionnaires. This was also useful for illiterate patients.

Questionnaire was administered at baseline before surgery and thereafter at 3, 6 and 12 months.

### Surgical procedure and data

Details of our operative and follow-up protocols were recorded in our previous publication [15]. Information on baseline characteristics, surgical procedures, complications and recurrence was obtained from our prospectively maintained database. We also collected data on the presence of stoma at various time-points. Morbidity was evaluated using common terminology criteria for adverse events version 3.0 of National Institute Health criteria.

### Statistical analysis

Disease-free survival (DFS) was defined as time from CRS and HIPEC procedure to disease recurrence or death from disease. OS was defined as time from the first CRS and HIPEC procedure to death. In the event that multiple CRS and HIPEC procedures were performed on an individual patient, OS was calculated from the first CRS and HIPEC.

QLQ-C30 and QLQ-CR29 scores were derived according to EORTC scoring manual [16]. Function and symptom summary scores were derived from the mean of individual scores.

Subscale and summary scores were summarised by time-point. Change from baseline scores was derived and analysed using a repeated measures model adjusting for time and baseline-by-time interaction. An unstructured covariance matrix was used in the model.

To assess the association between QoL and selected clinical variables, global health status score, function summary score and symptom summary score were summarised by time-point for each category of each variable.

We also performed an analysis of patients who underwent CRS and HIPEC procedures that had QoL questionnaires completed at all four time-points (completers only analysis). Completers were defined as patients with QoL questionnaires completed at all four time-points (baseline, 3 months, 6 months and 12 months post-op). Non-completers had procedures that were performed more than one year before data cut-off date of 18 Apr 2017 (window of 30 days given), but did not have four questionnaires completed.

The following procedures were excluded from analysis:

– Procedures which were performed less than one year (+30 days window) before data cut-off date of 18 Apr 2017 (i.e. not reached one year mark yet).
– If a patient completed questionnaires for all four time-points, but one or more of these were completed outside time window of ±1 month (i.e. 3 ± 1 months, 6 ± 1 months and 12 ± 1 months), then the procedure was taken as a completer, but questionnaires that were taken outside time window were excluded from analysis.

The association between ECOG performance status and morbidity or major morbidity was assessed using Pearson’s chi-squared test or Fisher’s exact test, where appropriate. The association between baseline QoL and morbidity or major morbidity was assessed using Wilcoxon rank-sum test. Only patients who did not have any previous CRS and HIPEC procedures were included in analysis. OS rates by ECOG performance status were estimated using Kaplan–Meier method. The association between ECOG performance status, baseline QoL and OS was assessed using Cox proportional hazards model. The association between QoL and death within one year was assessed using Wilcoxon rank-sum test for continuous variables and Fisher’s exact test for categorical variables.

Two-sided p-value of less than 0.05 was taken as statistically significant. Analyses were performed in Stata 15.0 (StataCorp, Texas, USA) and SAS 9.4 (SAS institute, Cary, NC, USA).

## Results

### Participants characteristics

Table 1 shows patients’ characteristics. One hundred and fifty one patients underwent 155 surgeries. Four patients underwent two surgeries. In all the analyses we treat patients who underwent two surgeries as two patients. We do not account for the fact that they are actually the same patient. Among four patients who underwent two surgeries, one patient had no stoma at the baseline of the first surgery but had the stoma at the baseline of the second surgery; another patient had the stoma at the baseline of the first surgery but had no stoma at the baseline of the second surgery. The number of patients with a stoma at baseline was 50.

**Table 1:** Patient demographics and surgical characteristics of patients who underwent CRS and HIPEC, n=151.

Characteristic		Frequency, n	Percentage, %
Total number of patients		151	
Total number of CRS and HIPEC procedures		155	100
	First CRS and HIPEC	143	92.3
	Second CRS and HIPEC	11	7.1
	Third CRS and HIPEC	1	0.6
Age at CRS and HIPEC, years	Median (range)	54 (19–76)	
Sex	Female	109	70.3
	Male	46	29.7
Primary tumour	Colorectal	50	32.3
	Ovarian	37	23.9
	Primary peritoneal	12	7.7
	Appendix	37	23.9
	Mesothelioma	6	3.9
	Others	13	8.4
Primary or recurrent tumour	Primary	58	37.4
	Recurrent	97	62.6
PCI score	Median (range)	10.5 (0–39)	
	0–14	99	63.9
	15–39	55	35.5
	Not available	1	0.6
Peritonectomy procedures	Omentectomy	120	77.4
	Left diaphragmatic stripping	49	31.6
	Right diaphragmatic stripping	71	45.8
	Left paracolic gutter	82	52.9
	Right paracolic gutter	89	57.4
	Pelvic peritoneum	106	68.4
	Liver capsule	55	35.5
	Small bowel mesentery	81	52.3
Additional procedures	Right (hemi)colectomy	51	32.9
	Left (hemi)colectomy	11	7.1
	Anterior resection	46	29.7
	Small bowel resection	43	27.7
	Bladder resection	7	4.5
	Ureterectomy	5	3.2
	Cholecystectomy	41	26.5
	Splenectomy	29	18.7
	Gastrectomy	10	6.5
	Pancreatectomy	2	1.3
	Salpingectomy	0	0
	Salpingoophorectomy	10	6.5
	THBSO	28	18.1
	Lymphadenectomy	6	3.9
	Others^b^	30	19.4
	Subtotal colectomy	5	3.2
	Appendicectomy	15	9.7
Duration of peritonectomy, min	Median (range)	430 (200–830)	
	Unknown	2	1.3
CC score	0	129	83.2
	1	24	15.5
	2	1	0.6
	Unknown	1	0.6
Length of SICU stay, days	Median (range)	1 (0–40)	
Length of hospital stay, days	Median (range)	13 (7–94)	
Post-operative complications	No	69	44.5
	Yes	86	55.5
High grade post-operative complications (Grade III and above)	No	134	86.5
	Yes	21	13.5
Post-operative complications^a^	Grade I	27	17.4
	Grade II	39	25.2
	Grade III	11	7.1
	Grade IV	10	6.5
	Grade V	0	0
Post-CRS and HIPEC adjuvant therapy	None	105	67.7
	Chemotherapy	47	30.3
	Radiotherapy	2	1.3
	Chemotherapy and radiotherapy	1	0.6

CRS, cytoreductive surgery; HIPEC, hyperthermic intraperitoneal chemotherapy; PCI, peritoneal carcinomatosis index; CC, completeness of cytoreduction; SICU, surgical intensive care unit; THBSO, total hysterectomy and bilateral salpingo-oophorectomy. This table summarises characteristics by CRS and HIPEC procedure. Some patients had multiple procedures, but were at least one year apart. ^a^Each procedure can have more than one complication. ^b^Some of the subtotal colectomy and appendicectomy cases have also been included in “Others”. The data needs to be reviewed and cleaned up.

A total of 472 QoL questionnaires were completed. Questionnaire completion was 80.0% at 3 months, 69.7% at 6 months and 54.8% at 12 months. A total of 4.5%, 9.7% and 20.6% of procedures had not reached 3 month, 6 month and 12 month time-point at the time of data cut-off, respectively.

Attrition due to death was 0.7% at 3 months, 3.6% at 6 months and 7.3% at 12 months. Procedures where 3, 6 or 12 month time-points were not reached yet were excluded from denominator. Questionnaires that were completed outside the allowed time window of ±1 month were considered attrition and counted under “Attrition due to other reasons”.

About 98.7% of patients had a completeness of cytoreduction (CC) score of 0 or 1.69 (44.5%) patients had no post-operative complications. Of 86 patients that suffered complications, only 21 (13.5%) had grade 3 or 4 complications while the remaining 86.5% had grade 1 or 2 complications.

The median follow-up duration was 18.9 months (95%CI, 15.0–20.7). Median DFS was 16.5 months (95%CI, 13.8–22.2). Median OS was not reached. Three year DFS and OS were 24.0% (95%CI, 14.9–34.3) and 73.0% (95%CI, 58.5–83.1), respectively.

### QoL scores change after CRS and HIPEC

**Figure 1:**
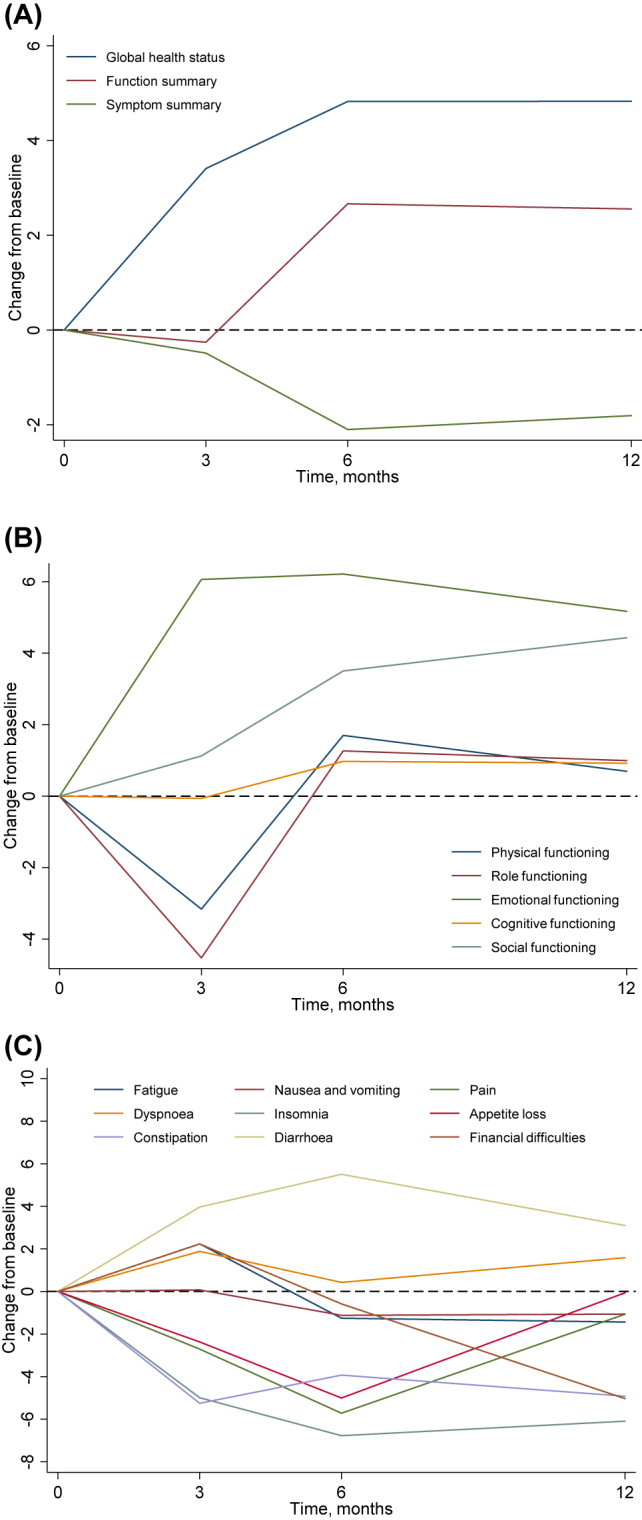
Plots of adjusted mean change from baseline-by-time for (A) global health status, functional scale scores and symptom scale scores, (B) functional scale scores, and (C) symptom scale scores. Global health status score significantly increased at 3, 6 and 12 months after CRS and HIPEC. The decreases in functional scales recovered to baseline by one year post-surgery. At 12 months, both emotional and social functioning scores improved compared to baseline. Insomnia and constipation symptoms were improved and more or less sustained up to 12 months. Diarrhoea symptoms increased.

Global health status score significantly increased at 3, 6 and 12 months after CRS and HIPEC (Figure 1A). At 12 months, the score increased by an average of 4.8 points (95%CI, 1.0–8.7; p=0.015) compared to baseline.

For functional scales (Figure 1B), the largest increase was observed in emotional functioning at 3 months after CRS and HIPEC (+6.1 points; 95%CI, 3.8–8.4; p<0.001), which was sustained at 6 and 12 months. Decreases in physical and role functioning was observed at 3 months, but had recovered by 6 months. At 12 months, both emotional and social functioning scores improved compared to baseline.

For symptom scales (Figure 1C), the largest post-operative improvements at 3 months were observed in insomnia (−5.0 points; 95% CI, from −8.4 to −1.6; p=0.005) and constipation (−5.3 points; 95%CI, from −8.0 to −2.5; p<0.001) symptoms, which were more or less sustained up to 12 months. However, diarrhoea symptoms increased (+4.0 points; 95%CI, 1.0–6.9; p=0.009).

One hundred and two CRS and HIPEC procedures (performed on 100 patients) with QoL questionnaires were completed at all four time-points. Thirteen procedures were classified as non-completers. Of these 13 procedures, four had baseline QoL only (no post-operative QoL data available). Thirty two procedures were performed within one year (+30 days window) of the data cut-off date, hence had not reached one year time-point yet. For eight procedures, patient died within one year. These 40 procedures were excluded from completers vs. non-completers analysis. The results were mostly similar to the analysis with all subjects. There appeared to be a larger increase in global health status and functional scale scores at 6 months for completers.

### The association between clinical variables and QoL scores

The following groups of patients had significantly lower global health status scores at 6 months compared to other patients at the same time-point (Table 2A): PCI score ≥15, SICU stay ≥1 day, presence of post-operative complications and patients who died within one year. Every 10 min increase in duration of peritonectomy was associated with a decrease of 0.28 points on average in global health status score at post-operative 6 months (95% CI, from −0.51 to −0.05; p=0.016) (Table 2B). By 12 months, the differences in scores between groups were no longer significant.

**Table 2:** (A) The association between clinical factors and QoL scores. (B) The association between the duration of peritonectomy, hospital stay and QoL scores. Peritoneal carcinomatosis index, presence of stoma, peritonectomy duration, death within one year, post-operative complication and length of SICU stay negatively influenced QoL.

(A)												
	Mean global health status score				Mean function summary score				Mean symptom summary score			
	Baseline	3 months	6 months	12 months	Baseline	3 months	6 months	12 months	Baseline	3 months	6 months	12 months
PCI score												
0–14	66.6	68.9	70.8	70.8	92.6	93.6	94.7	94.7	7.5	6.8	6.4	5.9
15–39	61.2	64.5	64.1	67.3	88.4	88.0	93.1	92.4	12.8	11.2	8.2	11.1
Post-operative complications												
No	68.5	69.9	73.5	71.1	92.7	94.9	96.0	95.3	6.4	5.4	4.5	5.0
Yes	61.1	65.5	64.2	69.0	89.7	89.1	92.6	93.0	12.2	10.6	9.2	9.5
Primary tumour												
Colorectal	65.5	66.9	71.1	70.4	89.9	90.4	93.0	93.8	10.0	7.6	6.4	6.9
Ovarian	66.9	67.6	64.7	71.9	88.0	88.4	91.3	92.9	10.2	11.1	11.1	7.6
Primary peritoneal	55.6	64.2	70.8	77.1	95.3	95.5	97.7	96.0	8.8	5.9	6.2	6.1
Appendix	64.2	72.3	71.3	62.3	93.1	93.9	97.5	93.4	9.1	7.2	4.0	9.4
Mesothelioma	62.5	75.0	58.3	62.5	86.2	81.4	94.7	93.5	16.3	16.3	10.5	13.3
Others	63.5	56.9	63.9	80.6	96.5	96.5	93.9	97.6	5.9	7.6	6.5	3.9
Stoma at baseline												
No	65.9	68.6	69.5	70.6	92.8	94.1	94.9	94.8	7.3	6.8	5.7	5.2
Yes	61.3	64.6	66.1	68.3	87.3	85.6	92.3	91.9	14.5	12.1	10.3	13.1
Recurrence within one year												
No	63.6	68.6	69.4	70.7	89.8	92.4	94.8	94.3	11.8	8.4	7.3	7.9
Yes	68.0	67.7	68.9	70.8	90.1	88.3	92.5	91.5	8.9	9.3	8.7	8.5
Death within one year												
No	65.6	69.6	70.2	69.9	90.5	90.7	94.6	94.0	10.3	8.9	7.4	7.5
Yes	60.2	38.3	25.0		83.7	72.4	64.7		19.8	24.6	37.7	

(B)								
Variable	Unit increase	Variables	3 months		6 months		12 months	
			Coefficient (95% CI)	p-Value	Coefficient (95% CI)	p-Value	Coefficient (95% CI)	p-Value
Duration of peritonectomy, min	10 min	Global health status score	−0.04 (−0.25, 0.18)	0.722	−0.28 (−0.51, −0.05)	0.016	0.02 (−0.26, 0.29)	0.911
		Function summary score	−0.19 (−0.35, −0.04)	0.015	−0.07 (−0.21, 0.06)	0.288	−0.04 (−0.15, 0.08)	0.533
		Symptom summary score	0.10 (−0.04, 0.25)	0.168	0.131 (−0.001, 0.264)	0.053	0.09 (−0.05, 0.23)	0.203
Hospital stay, days	One day	Global health status score	−0.004 (−0.236, 0.228)	0.971	−0.30 (−0.61, 0.02)	0.066	0.18 (−0.12, 0.47)	0.237
		Function summary score	−0.09 (−0.26, 0.09)	0.322	−0.04 (−0.23, 0.14)	0.635	−0.02 (−0.15, 0.10)	0.722
		Symptom summary score	0.04 (−0.12, 0.19)	0.645	0.09 (−0.09, 0.28)	0.307	0.14 (−0.01, 0.29)	0.069

CI, confidence interval. Global health status scores, function summary scores, symptom summary scores were analysed using a repeated measures model adjusting for time, baseline-by-time and variable-by-time interactions.

The following groups of patients had significantly lower function summary scores at post-operative 3 months, compared to other patients at the same time-point (Table 2A): presence of stoma at baseline and patients who died within one year. Every 10 min increase in duration of peritonectomy was associated with a decrease of 0.19 points on average in function summary score at post-operative 3 months (95%CI, from −0.35 to −0.04; p=0.015) (Table 2B). The differences observed in peritonectomy duration and presence of stoma were no longer significant at post-operative 6 months.

The following groups of patients had significantly higher symptom summary scores at post-operative 6 months, compared to other patients at the same time-point (Table 2A): SICU stay ≥1 day and patients who died within one year. The differences observed in SICU stay were no longer significant at 12 months post-op. The following groups of patients had significantly higher symptom summary scores at post-operative 12 months compared to other patients at the same time-point: PCI score ≥15 and presence of stoma at baseline.

At baseline, patients who died within one year tended to have higher appetite loss (p=0.030) and diarrhoea (p=0.026) scores compared to patients who were still alive at one year (Table 3). Primary tumour types distribution was significantly different between two groups (p=0.027). There was a stark contrast between two groups in global health status (p=0.001), physical functioning (p=0.023), role functioning (p=0.034), emotional functioning (p=0.037), fatigue (p=0.005), pain (p=0.025), appetite loss (p=0.017), symptom summary (p=0.034), nausea and vomiting (p=0.005) at three months; global health status (p=0.002), physical functioning (p=0.001), role functioning (p=0.008), social functioning (p=0.020), function summary (p=0.003), fatigue (p=0.022), pain (p=0.037), dyspnoea (p=0.018), appetite loss (p=0.018), symptom summary (p=0.004), nausea and vomiting (p=0.001) at six months.

**Table 3:** Comparison of patient demographics, surgical characteristics and baseline, 3, 6 and 12 months QoL scores between procedures where patients died within one year and those where patients survived more than one year. At baseline, patients who died within one year had higher appetite loss and diarrhoea scores compared to patients who were still alive at one year. Primary tumour types distribution was significantly different between two groups. There was a stark contrast between two groups in global health status, physical functioning, role functioning, emotional functioning, fatigue, pain, appetite loss, symptom summary, nausea and vomiting at three months; global health status, physical functioning, role functioning, social functioning, function summary, fatigue, pain, dyspnoea, appetite loss, symptom summary, nausea and vomiting at 6 months.

Variable	Total, n (%) or median (IQR)	Survived one year or more, n (%) or median (IQR)	Died within one year, n (%) or median (IQR)	Censored before one year, n (%) or median (IQR)	p-Value
Total number of patients	151				
Total number of CRS and HIPEC procedures	155	96	9	50	
Age at CRS and HIPEC, years	54 (46–62)	53 (45–59)	60 (56–62)	55.5 (50–63)	0.086
Sex					0.716
Female	109 (70.3%)	65 (67.7%)	7 (77.8%)	37 (74.0%)	
Male	46 (29.7%)	31 (32.3%)	2 (22.2%)	13 (26.0%)	
Primary tumour					0.027
Colorectal	50 (32.3%)	24 (25.0%)	5 (55.6%)	21 (42.0%)	
Ovarian	37 (23.9%)	25 (26.0%)	1 (11.1%)	11 (22.0%)	
Primary peritoneal	12 (7.7%)	11 (11.5%)	0 (0%)	1 (2.0%)	
Appendix	37 (23.9%)	26 (27.1%)	0 (0%)	11 (22.0%)	
Mesothelioma	6 (3.9%)	2 (2.1%)	1 (11.1%)	3 (6.0%)	
Others	13 (8.4%)	8 (8.3%)	2 (22.2%)	3 (6.0%)	
Primary or recurrent tumour					1.000
Primary	58 (37.4%)	37 (38.5%)	3 (33.3%)	18 (36.0%)	
Recurrent	97 (62.6%)	59 (61.5%)	6 (66.7%)	32 (64.0%)	
PCI score	10.5 (5–18)	11 (5–19)	15 (7–21)	8.5 (5–16)	0.506
Duration of peritonectomy, min	430 (330–560)	435 (325–575)	500 (420–635)	425 (330–525)	0.132
CC score					0.409
0	129 (83.2%)	82 (85.4%)	7 (77.8%)	40 (80.0%)	
1	24 (15.5%)	12 (12.5%)	2 (22.2%)	10 (20.0%)	
2	1 (0.6%)	1 (1.0%)	0 (0%)	0 (0%)	
Unknown	1 (0.6%)	1 (1.0%)	0 (0%)	0 (0%)	
Length of SICU stay, days	1 (0–2)	1 (0–2)	1 (1–2)	0 (0–1)	0.290
Length of hospital stay, days	13 (10–17)	13 (10–16.5)	19 (13–22)	11 (9–17)	0.195
Post-operative complications					0.087
No	69 (44.5%)	40 (41.7%)	1 (11.1%)	28 (56.0%)	
Yes	86 (55.5%)	56 (58.3%)	8 (88.9%)	22 (44.0%)	
High grade post-operative complications (Grade III and above)					0.117
No	134 (86.5%)	84 (87.5%)	6 (66.7%)	44 (88.0%)	
Yes	21 (13.5%)	12 (12.5%)	3 (33.3%)	6 (12.0%)	
Post-CRS and HIPEC adjuvant therapy					0.155
No	105 (67.7%)	60 (62.5%)	8 (88.9%)	37 (74.0%)	
Yes	50 (32.3%)	36 (37.5%)	1 (11.1%)	13 (26.0%)	
ECOG performance status					0.067
0	136 (87.7%)	86 (89.6%)	6 (66.7%)	44 (88.0%)	
1	18 (11.6%)	9 (9.4%)	3 (33.3%)	6 (12.0%)	
Unknown	1 (0.6%)	1 (1.0%)	0 (0%)	0 (0%)	
Baseline QLQ-C30 functional scales					
Global health status	66.7 (54.2–66.7)	66.7 (58.3–75)	66.7 (33.3–66.7)	66.7 (66.7–66.7)	0.581
Physical functioning	100 (93.3–100)	100 (93.3–100)	86.7 (86.7–100)	100 (93.3–100)	0.085
Role functioning	100 (100–100)	100 (100–100)	100 (66.7–100)	100 (100–100)	0.081
Emotional functioning	100 (75–100)	91.7 (75–100)	83.3 (75–100)	100 (83.3–100)	0.367
Cognitive functioning	100 (83.3–100)	100 (83.3–100)	100 (83.3–100)	100 (100–100)	0.287
Social functioning	100 (83.3–100)	100 (83.3–100)	100 (50–100)	100 (100–100)	0.695
Function summary	96.7 (88–100)	95.2 (85.7–100)	91.7 (79–97.3)	100 (92.3–100)	0.368
Baseline QLQ-C30 symptom scales					
Fatigue	0 (0–22.2)	0 (0–22.2)	11.1 (0–33.3)	0 (0–11.1)	0.311
Nausea and vomiting	0 (0–0)	0 (0–0)	0 (0–0)	0 (0–0)	0.331
Pain	0 (0–16.7)	0 (0–16.7)	0 (0–50)	0 (0–16.7)	0.301
Dyspnoea	0 (0–0)	0 (0–0)	0 (0–33.3)	0 (0–0)	0.065
Insomnia	0 (0–33.3)	0 (0–33.3)	0 (0–33.3)	0 (0–0)	0.875
Appetite loss	0 (0–0)	0 (0–0)	33.3 (0–33.3)	0 (0–0)	0.030
Constipation	0 (0–0)	0 (0–0)	0 (0–33.3)	0 (0–0)	0.269
Diarrhoea	0 (0–0)	0 (0–0)	0 (0–33.3)	0 (0–0)	0.026
Financial difficulties	0 (0–33.3)	0 (0–33.3)	0 (0–0)	0 (0–33.3)	0.625
Symptom summary	5.6 (0–13.6)	7.4 (0–14.8)	7.4 (3.7–38.9)	1.9 (0–7.4)	0.216
Three month QLQ-C30 functional scales	n=124	n=84	n=5	n=35	
Global health status	66.7 (66.7–83.3)	66.7 (66.7–83.3)	41.7 (33.3–50)	66.7 (66.7–66.7)	0.001
Physical functioning	100 (86.7–100)	100 (83.3–100)	66.7 (46.7–66.7)	100 (93.3–100)	0.023
Role functioning	100 (83.3–100)	100 (83.3–100)	66.7 (66.7–83.3)	100 (100–100)	0.034
Emotional functioning	100 (91.7–100)	100 (87.5–100)	83.3 (50–91.7)	100 (100–100)	0.037
Cognitive functioning	100 (83.3–100)	100 (83.3–100)	100 (83.3–100)	100 (100–100)	1.000
Social functioning	100 (100–100)	100 (100–100)	66.7 (66.7–100)	100 (100–100)	0.091
Function summary	98.3 (88.2–100)	96.7 (85–100)	72.7 (70–85)	100 (97.3–100)	0.052
Three month QLQ-C30 symptom scales	n=124	n=84	n=5	n=35	
Fatigue	0 (0–22.2)	0 (0–22.2)	33.3 (33.3–44.4)	0 (0–11.1)	0.005
Nausea and vomiting	0 (0–0)	0 (0–0)	16.7 (0–33.3)	0 (0–0)	0.005
Pain	0 (0–16.7)	0 (0–16.7)	16.7 (16.7–33.3)	0 (0–0)	0.025
Dyspnoea	0 (0–0)	0 (0–0)	0 (0–0)	0 (0–0)	1.000
Insomnia	0 (0–0)	0 (0–16.7)	33.3 (0–33.3)	0 (0–0)	0.182
Appetite loss	0 (0–0)	0 (0–0)	33.3 (0–33.3)	0 (0–0)	0.017
Constipation	0 (0–0)	0 (0–0)	0 (0–0)	0 (0–0)	0.180
Diarrhoea	0 (0–0)	0 (0–0)	0 (0–0)	0 (0–0)	1.000
Financial difficulties	0 (0–33.3)	0 (0–33.3)	0 (0–33.3)	0 (0–0)	0.841
Symptom summary	3.7 (0–11.1)	6.2 (0–11.4)	20.4 (8.6–24.1)	0 (0–4.9)	0.034
Six month QLQ-C30 functional scales	n=108	n=77	n=2	n=29	
Global health status	66.7 (66.7–83.3)	66.7 (66.7–83.3)	25 (16.7–33.3)	66.7 (66.7–66.7)	0.002
Physical functioning	100 (93.3–100)	100 (93.3–100)	56.7 (53.3–60)	100 (100–100)	0.001
Role functioning	100 (100–100)	100 (100–100)	50 (33.3–66.7)	100 (100–100)	0.008
Emotional functioning	100 (91.7–100)	100 (100–100)	75 (66.7–83.3)	100 (100–100)	0.075
Cognitive functioning	100 (100–100)	100 (100–100)	83.3 (83.3–83.3)	100 (100–100)	0.059
Social functioning	100 (100–100)	100 (100–100)	58.3 (50–66.7)	100 (100–100)	0.020
Function summary	100 (93.3–100)	98.7 (93.3–100)	64.7 (60.7–68.7)	100 (96.7–100)	0.003
Six month QLQ-C30 symptom scales	n=108	n=77	n=2	n=29	
Fatigue	0 (0–11.1)	0 (0–11.1)	55.6 (33.3–77.8)	0 (0–11.1)	0.022
Nausea and vomiting	0 (0–0)	0 (0–0)	41.7 (33.3–50)	0 (0–0)	0.001
Pain	0 (0–0)	0 (0–0)	25 (16.7–33.3)	0 (0–16.7)	0.037
Dyspnoea	0 (0–0)	0 (0–0)	50 (33.3–66.7)	0 (0–0)	0.018
Insomnia	0 (0–0)	0 (0–0)	33.3 (33.3–33.3)	0 (0–0)	0.123
Appetite loss	0 (0–0)	0 (0–0)	33.3 (33.3–33.3)	0 (0–0)	0.018
Constipation	0 (0–0)	0 (0–0)	16.7 (0–33.3)	0 (0–0)	0.608
Diarrhoea	0 (0–0)	0 (0–0)	33.3 (0–66.7)	0 (0–0)	0.302
Financial difficulties	0 (0–33.3)	0 (0–33.3)	50 (33.3–66.7)	0 (0–0)	0.165
Symptom summary	3.1 (0–11.1)	3.7 (0–11.1)	37.7 (29.6–45.7)	0 (0–4.9)	0.004
12 month QLQ-C30 functional scales	n=85	n=73	n=0	n=12	
Global health status	66.7 (66.7–83.3)	66.7 (66.7–83.3)		66.7 (66.7–91.7)	NA
Physical functioning	100 (93.3–100)	100 (93.3–100)		100 (93.3–100)	NA
Role functioning	100 (100–100)	100 (100–100)		100 (100–100)	NA
Emotional functioning	100 (83.3–100)	100 (91.7–100)		100 (75–100)	NA
Cognitive functioning	100 (100–100)	100 (100–100)		100 (91.7–100)	NA
Social functioning	100 (100–100)	100 (100–100)		100 (100–100)	NA
Function summary	96.7 (90–100)	97 (90.3–100)		96.3 (86–100)	NA
12 month QLQ-C30 symptom scales	n=85	n=73	n=0	n=12	
Fatigue	0 (0–22.2)	0 (0–11.1)		5.6 (0–22.2)	NA
Nausea and vomiting	0 (0–0)	0 (0–0)		0 (0–0)	NA
Pain	0 (0–16.7)	0 (0–16.7)		8.3 (0–25)	NA
Dyspnoea	0 (0–0)	0 (0–0)		0 (0–0)	NA
Insomnia	0 (0–33.3)	0 (0–33.3)		0 (0–16.7)	NA
Appetite loss	0 (0–0)	0 (0–0)		0 (0–16.7)	NA
Constipation	0 (0–0)	0 (0–0)		0 (0–0)	NA
Diarrhoea	0 (0–0)	0 (0–0)		0 (0–0)	NA
Financial difficulties	0 (0–0)	0 (0–0)		0 (0–0)	NA
Symptom summary	3.7 (0–12.0)	3.7 (0–13.0)		4.9 (0–9.3)	NA

This Table summarises characteristics by CRS and HIPEC procedure. Some patients had multiple procedures, but were at least one year apart. Procedures where the patient was censored before one year were excluded from the comparison. IQR, interquartile range; n, number of observations.

### The association between ECOG performance status, QoL at baseline and morbidity

All patients had an ECOG performance status of either 0 or 1 at baseline (Table 4). Total morbidity rate was 55.5%. Major morbidity rate was 13.5%. Patients with ECOG performance status of 1 at baseline were more likely to have major morbidity (27.8% vs. 11.8%), although not statistical significance (p=0.075).

**Table 4:** Association between ECOG performance status, QoL at baseline and morbidity. Morbidity was associated with significantly lower global health status, physical functioning and role functioning scores, and significantly higher symptom summary score, fatigue, nausea and vomiting, dyspnoea, appetite loss, constipation and financial difficulties scores at baseline. Major morbidity was associated with significantly lower global health status and social functioning scores, and significantly higher symptom summary score, fatigue and financial difficulties scores at baseline.

Characteristic		No morbidity, n (%) or mean ± SD	Morbidity, n (%) or mean ± SD	p-Value	No major morbidity, n (%) or mean ± SD	Major morbidity, n (%) or mean ± SD	p-Value
ECOG performance status				0.974			0.075
	0	61 (44.9%)	75 (55.1%)		120 (88.2%)	16 (11.8%)	
	1	8 (44.4%)	10 (55.6%)		13 (72.2%)	5 (27.8%)	
	Unknown	0 (0%)	1 (100%)		1 (100%)	0 (0%)	
QLQ-C30 functional scales							
	Global health status	68.5 ± 16.5	61.1 ± 19.6	0.037	65.5 ± 18.7	57.5 ± 16.9	0.022
	Physical functioning	95.7 ± 8.9	89.9 ± 17.1	0.021	92.4 ± 14.4	93.0 ± 13.9	0.953
	Role functioning	96.6 ± 12.0	89.7 ± 21.4	0.015	93.3 ± 17.9	89.7 ± 19.3	0.337
	Emotional functioning	85.5 ± 21.5	88.3 ± 16.4	0.986	87.1 ± 18.5	86.9 ± 21.2	0.809
	Cognitive functioning	93.5 ± 13.2	94.0 ± 12.8	0.705	93.4 ± 13.4	96.0 ± 9.0	0.44
	Social functioning	92.3 ± 19.7	86.4 ± 23.6	0.093	90.5 ± 20.9	79.4 ± 27.3	0.047
	Function summary	92.7 ± 11.4	89.7 ± 13.5	0.071	91.3 ± 12.4	89.0 ± 14.4	0.477
QLQ-C30 symptom scales							
	Fatigue	7.6 ± 14.9	15.6 ± 22.7	0.014	10.9 ± 19.6	19.6 ± 21.3	0.016
	Nausea and vomiting	0.7 ± 3.4	4.8 ± 14.7	0.039	2.4 ± 10.0	7.1 ± 17.1	0.102
	Pain	8.7 ± 16.6	14.0 ± 20.4	0.105	10.9 ± 18.5	15.9 ± 21.4	0.225
	Dyspnoea	2.9 ± 11.1	8.1 ± 16.9	0.018	6.0 ± 15.2	4.8 ± 12.0	0.899
	Insomnia	13.0 ± 23.7	16.3 ± 27.4	0.547	14.2 ± 26.0	19.0 ± 24.9	0.224
	Appetite loss	3.4 ± 11.6	13.6 ± 25.2	0.003	8.2 ± 20.6	14.3 ± 22.5	0.081
	Constipation	5.8 ± 16.1	11.6 ± 20.3	0.022	8.2 ± 18.0	14.3 ± 22.5	0.159
	Diarrhoea	3.4 ± 11.6	4.7 ± 15.5	0.702	3.5 ± 13.1	7.9 ± 18.0	0.117
	Financial difficulties	12.1 ± 24.2	21.3 ± 32.7	0.047	15.2 ± 28.2	30.2 ± 34.8	0.011
	Symptom summary	6.4 ± 9.2	12.2 ± 13.5	0.003	8.8 ± 11.8	14.8 ± 13.1	0.012

SD, standard deviation; ECOG, Eastern Cooperative Oncology Group.

Morbidity was associated with significantly lower global health status, physical functioning and role functioning scores, and significantly higher fatigue, nausea and vomiting, dyspnoea, appetite loss, constipation and financial difficulties scores at baseline. Symptom summary score at baseline was significantly higher in patients with morbidity.

Major morbidity was associated with significantly lower global health status and social functioning scores, and significantly higher fatigue and financial difficulties scores at baseline. Symptom summary score at baseline was also significantly higher in patients with major morbidity.

### The association between baseline QoL and ECOG performance status with OS

Twenty-three died in 143 patients (16.1%). As only one patient died within 30 days, the association between baseline QoL and ECOG performance status with 30 day mortality was not assessed. Six patients were lost to follow-up before 30 days. Therefore, 30 day mortality rate was 0.7% (1/149).

Although patients with ECOG performance status of 1 at baseline had lower 2-year survival rates (Table 5), this was not statistically significant (p=0.476). Note that survival curves were not proportional (Figure 1). This might be due to immature data, since only 16.1% events were observed.

**Table 5:** Association between ECOG performance status, QoL at baseline and overall survival, adjusted for primary tumour. Lower social functioning score, and higher pain, dyspnoea and symptom summary scores at baseline were significantly associated with poorer OS.

Characteristic	Unit increase	Adjusted hazard ratio (95% CI)	p-Value
ECOG performance status	NA		
0		1	
1		1.50 (0.49, 4.53)	0.476
QLQ-C30 functional scales			
Global health status	10	0.97 (0.77, 1.21)	0.766
Physical functioning	10	0.87 (0.67, 1.14)	0.314
Role functioning	10	0.90 (0.75, 1.07)	0.237
Emotional functioning	10	0.98 (0.82, 1.17)	0.822
Cognitive functioning	10	0.94 (0.70, 1.26)	0.674
Social functioning	10	0.81 (0.70, 0.94)	0.006
Function summary	10	0.72 (0.52, 1.00)	0.051
QLQ-C30 symptom scales			
Fatigue	10	1.18 (0.99, 1.41)	0.068
Nausea and vomiting	10	1.17 (0.91, 1.51)	0.215
Pain	10	1.24 (1.04, 1.49)	0.019
Dyspnoea	10	1.57 (1.27, 1.94)	<0.001
Insomnia	10	0.96 (0.83, 1.11)	0.573
Appetite loss	10	1.07 (0.90, 1.27)	0.430
Constipation	10	1.17 (0.99, 1.39)	0.058
Diarrhoea	10	0.92 (0.77, 1.11)	0.376
Financial difficulties	10	1.10 (0.98, 1.24)	0.117
Symptom summary	10	1.36 (1.01, 1.84)	0.041

The analysis was adjusted for primary tumour, fitted as a categorical variable comprising the following categories: 1) colorectal, 2) ovarian/primary peritoneal, 3) appendix, 4) others (note that hazard ratios for primary tumour are not shown in the Table).

Social functioning score at baseline was significantly associated with OS (Table 5). For every 10-unit increase in social functioning score, the hazard of death decreased by 19% (HR=0.81; 95%CI, 0.70–0.94; p=0.006).

Pain, dyspnoea and symptom summary scores at baseline were also significantly associated with OS (Table 5). A 10-unit increase in pain score was associated with a 24% increase in hazard of death (HR=1.24; 95%CI, 1.04–1.49; p=0.019). A 10-unit increase in dyspnoea score was associated with a 57% increase in hazard of death (HR=1.57; 95%CI, 1.27–1.94; p<0.001). A 10-unit increase in symptom summary score was associated with 36% increase in hazard of death (HR=1.36; 95%CI, 1.01–1.84; p=0.041).

## Discussion

Our study is one of the few prospective studies to assess the prognostic value of QoL in survivors of patients who underwent CRS and HIPEC for PC from various primary sites. It is also the largest prospective study done to date in Asia to assess the impact of CRS and HIPEC on QoL over time and determinants of post-operative QoL in this particular group of patients. This study echoes our previous publication [17] as well as the other studies mentioned above. Most studies showed an impairment of QoL immediately after surgery which continues at 3 months. However, scores usually return to baseline by 6–12 months [7]. We showed a significant increase in global health score to above baseline by 12 months, recovery of physical and role functions at 6 months and recovery of social functioning by 12 months. Emotional functioning recovered the fastest at 3 months, which is an indication of the benefit of the surgery at giving these patients a ray of hope when all else has failed and all others have written them off. We have previously attributed this finding to response shift, whereby patient’s emotional well-being is good despite physical and functional disabilities because of the knowledge that their cancer is removed and they have a chance at long-term survival.

Using QoL to predict morbidity, mortality and survival is also not a new concept, even in CRS and HIPEC. However, limited studies investigate the association between baseline QoL and post-operative outcomes in survivors of patients who underwent CRS and HIPEC for PC from various primary sites. Ihemelandu et al. showed better ECOG and better emotional well-being resulted in less complications [8]. The same group of authors also showed in a separate article [9] that a higher baseline FACT-General, FACT-C, physical well-being, trial outcome index, and SF-36 vitality were associated with improved survival. Higher baseline BPI, worst pain and ECOG were associated with worse survival. Our study showed lower global health, social functioning scores, symptom summary scores and financial difficulties at baseline were associated with major morbidity. Patients with ECOG performance status of 1 were also more likely to develop a major morbidity. Looking at OS, our results suggested patients with ECOG performance status of 1 were likely to have a lower 2-year OS. Higher social functioning scores, less pain, less dyspnoea and less symptom summary score were associated with higher OS. While patients who died within one year tended to have higher appetite loss scores (p=0.030) and diarrhoea scores (p=0.026) at baseline compared to patients who were still alive at 1-year, lower appetite loss and diarrhoea scores at baseline were not associated with higher OS (p=0.430 and p=0.376 respectively). Previously, a surgeon may have swayed the patient’s decision for CRS and HIPEC based on a general feel of the patient’s overall condition. By using a quantitative tool, such as baseline QoL, one can now counsel patients better on how their baseline status may affect their outcome.

We found PCI, presence of stoma and post-operative complication negatively influenced post-operative QoL, which echoes a recent systematic analysis [7]. We also found peritonectomy duration, death within one year and length of SICU stay adversely affect post-operative QoL. Patients who recurred within one year had very similar global health scores compared to patients who did not recur in one year. However, Passot G et al. [18] reported recurrent disease negatively influenced QoL at 12 months. These different results may be because we used different QoL questionnaires and patient ethics – our study used EORTC-QLQ-C30 in Asian patients while Passot G team used Gastro-Intestinal Quality of Life Index questionnaire in Western patients. Although our previous publication [19] reported patients without recurrences had higher global health score, it was not statistically significant. Moreover, it was a cross-sectional study and QoL was assessed at various time-points.

## Conclusions

Our study and previous ones suggest not only is QoL not adversely affected by CRS and HIPEC but may in effect, improve aspects of patient’s QoL (e.g. emotional functioning). Additionally, baseline QoL may play an important role in the selection of patients and should be taken into consideration with tumour and patient characteristics as it can affect and possibly predict for peri-operative morbidity and OS. It may not be used as a tool to decide if patient is suitable for surgery but it can help surgeon and patient make a more informed decision.
